# Inflammation and remodeling pathways and risk of cardiovascular events in patients with ischemic heart failure and reduced ejection fraction

**DOI:** 10.1038/s41598-022-12385-0

**Published:** 2022-05-20

**Authors:** Nicolas Girerd, John Cleland, Stefan D. Anker, William Byra, Carolyn S. P. Lam, David Lapolice, Mandeep R. Mehra, Dirk J. van Veldhuisen, Emmanuel Bresso, Zohra Lamiral, Barry Greenberg, Faiez Zannad

**Affiliations:** 1 Université de Lorraine, Centre d’Investigation Clinique-Plurithématique Inserm CIC-P 1433, Inserm U1116, CHRU Nancy Brabois, F-CRIN INI-CRCT (Cardiovascular and Renal Clinical Trialists), Nancy, France; 2 Robertson Centre for Biostatistics and Clinical Trials, University of Glasgow, Glasgow, Scotland; 3 Department of Cardiology (CVK), and Berlin Institute of Health Center for Regenerative Therapies (BCRT), German Centre for Cardiovascular Research (DZHK) Partner Site Berlin, Charité Universitätsmedizin Berlin, Berlin, Germany; 4 Janssen Research and Development, Raritan, NJ USA; 5 National Heart Centre Singapore, Duke-National University of Singapore, Singapore, Singapore; 6 Brigham and Women’s Hospital and Harvard Medical School, Boston, MA USA; 7 Department of Cardiology, University Medical Center Groningen, University of Groningen, Groningen, The Netherlands; 8 Cardiology Division, Department of Medicine, University of California, La Jolla, San Diego, USA

**Keywords:** Cardiology, Cardiovascular biology

## Abstract

Patients with heart failure (HF) and coronary artery disease (CAD) have a high risk for cardiovascular (CV) events including HF hospitalization, stroke, myocardial infarction (MI) and sudden cardiac death (SCD). The present study evaluated associations of proteomic biomarkers with CV outcome in patients with CAD and HF with reduced ejection fraction (HFrEF), shortly after a worsening HF episode. We performed a case–control study within the COMMANDER HF international, double-blind, randomized placebo-controlled trial investigating the effects of the factor-Xa inhibitor rivaroxaban. Patients with the following first clinical events: HF hospitalization, SCD and the composite of MI or stroke were matched with corresponding controls for age, sex and study drug. Plasma concentrations of 276 proteins with known associations with CV and cardiometabolic mechanisms were analyzed. Results were corrected for multiple testing using false discovery rate (FDR). In 485 cases and 455 controls, 49 proteins were significantly associated with clinical events of which seven had an adjusted FDR < 0.001 (NT-proBNP, BNP, T-cell immunoglobulin and mucin domain containing 4 (TIMD4), fibroblast growth factor 23 (FGF-23), growth differentiation factor-15 (GDF-15), pulmonary surfactant-associated protein D (PSP-D) and Spondin-1 (SPON1)). No significant interactions were identified between the type of clinical event (MI/stroke, SCD or HFH) and specific biomarkers (all interaction FDR > 0.20). When adding the biomarkers significantly associated with the above outcome to a clinical model (including NT-proBNP), the C-index increase was 0.057 (0.033–0.082), p < 0.0001 and the net reclassification index was 54.9 (42.5 to 67.3), p < 0.0001. In patients with HFrEF and CAD following HF hospitalization, we found that NT-proBNP, BNP, TIMD4, FGF-23, GDF-15, PSP-D and SPON1, biomarkers broadly associated with inflammation and remodeling mechanistic pathways, were strong but indiscriminate predictors of a variety of individual CV events.

## Introduction

Patients with heart failure (HF) and coronary artery disease (CAD) are at excess risk of cardiovascular (CV) events, including HF hospitalization, stroke and myocardial infarction (MI). Each of these individual events is associated with a three to fourfold increase in the subsequent risk of death, including sudden cardiac death (SCD), in patients with HF with reduced ejection fraction (HFrEF)^1^.

Proteomic analysis that explores serum protein components can provide insight into the mechanism of the multiple biological processes underlying disease progression^2^ and can be used to study mechanisms associated with various clinical events. By applying knowledge-based network analysis, associations among protein biomarkers (BMs) of pathophysiological importance can be discovered in patients with HF^3^ that would allow for the identification and understanding of new mechanistic pathways leading to clinical events and could ultimately provide insight into novel therapeutic strategies. Although proteomic marker discovery approaches have been shown to predict death^4,5^ and HF hospitalization^4^, their prognostic value in the specific setting of patients with CAD and HFrEF following HF hospitalization to predict other outcomes (such as stroke and MI) has been insufficiently explored. In addition, factors that determine whether these patients are more prone to develop a SCD, HF hospitalization, stroke or MI are unclear. Currently, few analyses exist regarding the prognostic value of proteomic profiles in the specific setting of worsening HF^4^. Determining the mechanistic bioprofiles of patients respectively at risk of SCD, HFH, stroke or MI, in the setting of worsening HF, may help more specific targeting of strategies to prevent such events in future trials. This enrichment may additionally help inform the design of acute HF trials to increase the likelihood of their conclusiveness, something that has been only rarely achieved over the past 40 years^6^.

The COMMANDER HF (Rivaroxaban in Patients with Heart Failure, Sinus Rhythm, and Coronary Disease) trial^7^ enrolled chronic CAD patients shortly following hospitalization for worsening HF. Blood samples were collected at the time of entry into the study and follow-up cardiovascular events were carefully recorded in the population as outlined in the study protocol. In the present study, we investigated whether analysis of baseline proteomics data helps predict subsequent CV events, including rehospitalization for HF, MI/stroke and SCD and whether proteomic analysis adds to the understanding of mechanisms underlying progression to these events following an admission for worsening HF.

## Methods

### Population

We performed a nested case–control analysis of the COMMANDER HF trial. Briefly, the COMMANDER HF trial included 5022 patients with worsening HF in the context of chronic HFrEF and underlying CAD^7–9^. All patients had a left ventricular ejection fraction (LVEF) ≤ 40%, CAD, and elevated plasma concentrations of natriuretic peptides (this latter criterion added during the course of the trial) and did not have atrial fibrillation. They were randomly assigned to receive rivaroxaban at a dose of 2.5 mg twice daily or placebo in addition to standard care. Over a median follow-up period of 21.1 months, the primary endpoint occurred in 626 (25.0%) of 2507 patients assigned to rivaroxaban and in 658 (26.2%) of 2515 patients assigned to placebo (hazard ratio, 0.94; 95% confidence interval [CI], 0.84 to 1.05; P = 0.27). This international trial was approved by all relevant committees in each country, and every patients provided written consent. All methods used followed relevant regulations.

### Outcomes

The primary efficacy outcome of the COMMANDER HF trial was the composite of death from any cause, MI or stroke. Three clinical events were considered in the present analysis, namely HF hospitalization, SCD and the composite of MI/stroke, as ischemic events. In addition, we also analyzed the composite of all events at once, i.e. HF hospitalization, SCD and MI/stroke.

### Matching procedure

Controls were matched for age, sex and study drug with cases that had one of the 4 clinical events described above (i.e. HF hospitalization, SCD and the composite of MI/ischemic stroke). Suitable patients could serve as controls for more than one case when no other suitable control could be identified. All of the cases that could be matched with a control and had available blood samples were considered in this analysis. Cases of a given event could experience another event during follow-up (e.g. a case patient for MI could subsequently experience stroke) (supplementary Table 1).

### Sample handling

All sample shipments and sample data acquisition within the COMMANDER HF trial were carried out according to predefined standard operating procedures and material transfer agreements to maintain uniformity. The cases and controls were separately identified and selected. All participants’ information was then removed and a randomly-sorted list of patient/sample IDs for each cohort was sent to the bioassay facility. The entire sampling handling/protein measurement procedure was carried out while maintaining the blind.

### Assays and studied biomarkers

Baseline plasma samples were analyzed for protein biomarkers using the OLINK Proseek® Multiplex cardiovascular (CVD) II, CVD III, and cardiometabolic (CM) panels. These panels were selected for the well-balanced inclusion of proteins with established or likely associations with CV disease (the full information on these panels is available at: https://www.olink.com/resources-support/document-download-center/). The assays use a proximity extension assay (PEA) technology, where 92 oligonucleotide-labeled antibody probe pairs per panel are allowed to bind to their respective targets in the sample in 96-well plate format. When binding to their correct targets, they give rise to new DNA amplicons with each ID-barcoding their respective antigens. The amplicons are subsequently quantified using a Fluidigm BioMark™ HD real-time PCR platform. The OLINK platform provides log2-normalized protein expression (NPX) data. The Olink^®^ quality control samples are considered as “flagged” if they deviate more than 0.3 NPX from the median of all samples in one of two control assays for incubation and detection. The LOD is defined by the three negative controls run on each plate and set to three standard deviations above the measured background.

The assays were performed “blinded” to case/control status with cases and controls randomly distributed across plates. The proteomic results were then merged with the baseline data, which included the case–control status, matching variables and the clinical risk factors. A total of 276 proteins were ultimately analyzed.

### Statistical analysis

For baseline clinical characteristics, continuous variables are expressed as means ± standard deviation (SD) or as medians (interquartile range) if the distribution was skewed and categorical variables as frequencies and percentages. Baseline characteristics of participants were compared between cases and controls using Fisher’s exact test for categorical variables and t-tests or non-parametric Wilcoxon tests for continuous variables, as appropriate.

To assess the association of biomarkers with outcomes, we performed logistic regression models adjusted for age, sex and factors significantly associated with case/control status (as reported in Table 1). The association with event risk was assessed for each biomarker individually (mono-marker models). These associations were first assessed for the composite outcome of hospitalization for worsening HF (WHF), sudden death and MI/stroke and subsequently for “individual” outcomes (either hospitalization for WHF, sudden death or MI/stroke). In addition, we searched for an interaction between biomarkers and the type of clinical event (either hospitalization for WHF, sudden death or MI/stroke). To achieve this, an interaction term was introduced between each biomarker and the outcome subgroup; this interaction term tests whether the association of biomarkers with outcome is homogenous across outcome types. In an additional analysis, the models were further adjusted for NT-proBNP. No imputation was performed during the analysis process.

**Table 1:** Patient characteristics according to MI/stroke, SCD, HF rehospitalization overall in the sex and age-matched COMMANDER HF population.

Characteristic	All events			
	Controls	Cases	SMD (%)	p
	(n = 455)	(n = 485)		
Age (yrs)	67.4 ± 10.0	67.3 ± 10.3	1.40	0.83
Study drug	210 (46.2%)	230 (47.3%)	2.30	0.74
Female sex	110 (24.2%)	120 (24.7%)	1.20	0.88
**Race**			14.60	0.75
White	405 (89.0%)	433 (89.1%)		
Black	2 (0.4%)	5 (1.0%)		
Asian	42 (9.2%)	41 (8.4%)		
Other	6 (1.3%)	7 (1.4%)		
**Region**			21.50	0.13
Eastern Europe	315 (69.2%)	332 (68.3%)		
North America	5 (1.1%)	9 (1.9%)		
Asia Pacific	42 (9.2%)	41 (8.4%)		
Latin America	50 (11.0%)	38 (7.8%)		
Western Europe	43 (9.5%)	66 (13.6%)		
BMI (kg/m^2^)	27.6 ± 5.0	27.9 ± 5.1	6.30	0.33
eGFR (ml/min/1.73 m^2^)	**70.1 ± 22.9**	**64.7 ± 23.0**	**23.80**	**0.0003**
**eGFR (ml/min/1.73 m**^**2**^**)**			**23.60**	**0.0051**
< 30 ml/min/1.73 m^2^	11 (2.4%)	25 (5.1%)		
30 to < 60 ml/min/1.73 m^2^	157 (34.5%)	199 (40.9%)		
60 to < 90 ml/min/1.73 m^2^	204 (44.8%)	201 (41.4%)		
≥ 90 ml/min/1.73 m^2^	83 (18.2%)	61 (12.6%)		
BNP level (pg/ml)	875.2 ± 787.4	950.3 ± 727.8	9.90	0.53
Log2 BNP (pg/ml)	6.4 ± 0.8	6.6 ± 0.7	21.70	0.17
BNP rank (pg/ml)				
NT-proBNP (pg/ml)	5869 ± 8707	5568 ± 7310	3.70	0.66
Log2 NT-proBNP (pg/ml)				
NT-proBNP rank	265.8 ± 160.5	281.2 ± 154.9	9.80	0.26
d-dimer level (mg/l)	637.2 ± 1012.5	732.3 ± 1027.4	9.30	0.16
Log2 d-dimer	6.0 ± 0.9	6.2 ± 0.8	24.40	0.0002
d-dimer rank				
Ejection fraction (%)	**33.1 ± 6.7**	**31.1 ± 7.3**	**28.40**	** < 0.0001**
Ejection fraction rank	**511.5 ± 267.6**	**433.0 ± 269.1**	**29.30**	** < 0.0001**
**NYHA**			**12.40**	**0.031**
I/II	**238 (52.4%)**	**226 (46.5%)**		
III	**214 (47.0%)**	**246 (50.6%)**		
IV	**3 (0.7%)**	**14 (2.9%)**		
Myocardial infarction	**331 (72.7%)**	**385 (79.2%)**	**15.20**	**0.022**
Stroke	44 (9.7%)	58 (11.9%)	7.30	0.29
Diabetes	**163 (35.8%)**	**223 (45.9%)**	**20.60**	**0.002**
Hypertension	339 (74.5%)	375 (77.2%)	6.20	0.36
ACEI or ARB	431 (94.7%)	452 (93.0%)	7.20	0.28
Beta blockers	424 (93.2%)	445 (91.6%)	6.10	0.39
MRA	**327 (71.9%)**	**383 (78.8%)**	**16.10**	**0.015**
Digoxin	**20 (4.4%)**	**43 (8.8%)**	**18.00**	**0.009**
Aspirin	426 (93.6%)	450 (92.6%)	4.10	0.61

Significant values Queryare given in bold.

In all 3 cohorts, analysis was corrected for multiple testing using a false discovery rate (FDR) of 5%, applying the Benjamini–Hochberg procedure^10^.

Since proteins were measured using NPX (Normalized Protein eXpression) values (details regarding NPX values can be found at: https://www.olink.com/question/what-is-npx/), the odds ratio for each protein estimates the increase in the odds of HF associated with a doubling in protein concentration.

In a supplementary analysis, we used a stabilized inverse probability of treatment weighting method (sIPTW) based on propensity score^11^. Weights were obtained from a logistic regression with outcome as dependent variable and clinical adjustment variables as explanatory variables (sex, age, study drug and significant factors from Table 1 (eGFR, LVEF, NYHA class, MI, diabetes, MRA and digoxin).

To determine the added predictive value of biomarkers on top on clinical predictors, we assessed the C-index of a baseline model including all adjustment variables (matching variables [sex, age, study drug] and variables significantly different across groups in Table 1 [namely eGFR, LVEF, NYHA class, MI, diabetes, MRA and digoxin]) and assessed the increase in C-index when adding biomarkers to the model. In addition, the increase in discriminative value of the addition of biomarkers on top of the aforementioned clinical model was assessed using continuous net reclassification improvement (NRI)^12^.

### Complex network analysis

Pathway overrepresentation was calculated for proteins selected by the statistical analysis. Pathway-annotation proteins were extracted from the FHF-GKBox^13^. In the FHF-GKBox, protein-pathway relationships were extracted from Reactome (version 69)^14^. The entire FHF-GKBox was considered as annotation background for a Fisher’s exact test and obtained p-values were corrected for multiple testing using a FDR of 5% by applying the Benjamini–Hochberg method. Only pathways with a FDR lower than 5% and selected proteins linked to these pathways were considered to construct the graph. The network was displayed using Cytoscape (version 3.7.2)^15^.

## Results

### Patient characteristics

The analysis included 485 cases and 455 controls (as a subject could be a control for more than one case). Mean age was 67 years and 24% of patients were female (Table 1). Patients with clinical events had significantly lower eGFR and LVEF than controls (Table 1). They also had a higher probability of MI history and diabetes and were more likely to be treated with MRA or digoxin.

Overall, the characteristics according to the presence/absence of specific events (MI/stroke, SCD or HFH) were similar to those observed in the complete case/control study (Supplementary Table 2).

### Association of protein biomarkers with total cardiovascular clinical events

**Figure 1:**
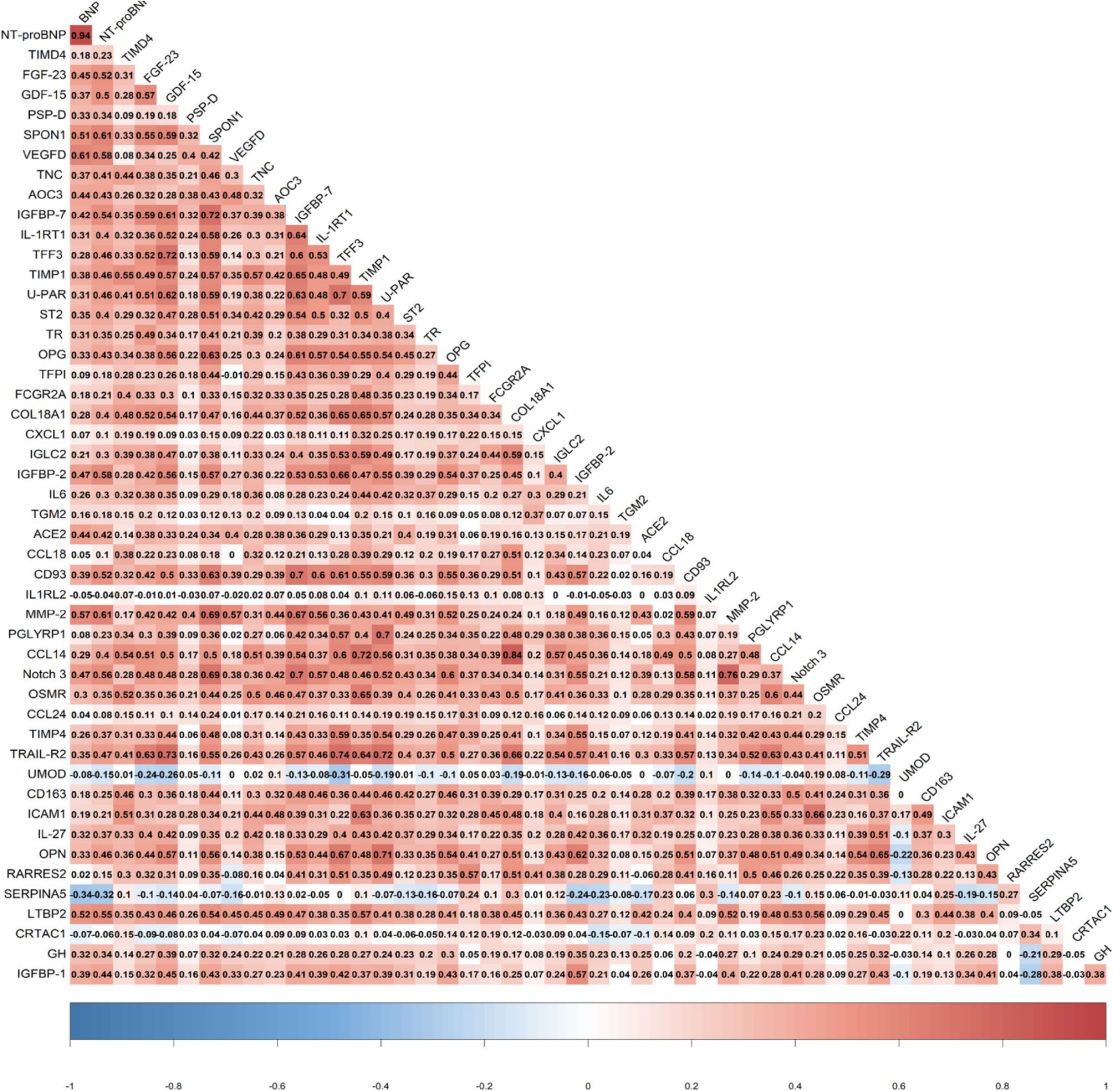
Heat map of biomarkers identified as significantly associated with events (as in Table 2).

When correcting for the multiplicity of tests and adjusting for age, sex, study drug and relevant factors identified in univariable analysis, 49 proteins were significantly associated with CV clinical events at the p < 0.05 FDR threshold. The 7 proteins associated with outcome with an FDR < 0.001 were NT-proBNP, BNP, T-cell immunoglobulin and mucin domain containing 4 (TIMD4), fibroblast growth factor 23 (FGF-23), growth differentiation factor-15 (GDF-15), pulmonary surfactant-associated protein D (PSP-D) and Spondin-1 (SPON1), all positively associated with a increased risk of CV events. These biomarkers, except for BNP and NT-proBNP, were poorly to moderately correlated with each other (Fig. 1).

When further adjusting for NT-proBNP, 11 proteins were still significantly associated with the composite CV outcome, namely TIMD4, CCL18, IL1RL2, CXCL1, FCGR2A, PSP-D, UMOD, CRTAC1, CCL24, TFPI and TGM2 (Table 2).

**Table 2:** Significant adjusted associations of protein biomarkers with CV events.

Biomarker	Models adjusted for clinical variables^a^		Models adjusted for clinical variables^a^ and NT-proBNP
	OR (95% CI)	FDR	OR (95% CI)
**NT-proBNP**	**1.376 (1.259–1.504)**	** < 0.0001**	**–**
**BNP**	**1.292 (1.202–1.390)**	** < 0.0001**	**–**
**TIMD4**	**1.723 (1.367–2.171)**	**0.0004**	**1.484 (1.169–1.884)**
**FGF-23**	**1.265 (1.141–1.402)**	**0.0005**	1.094 (0.978–1.224)
**GDF-15**	**1.514 (1.257–1.824)**	**0.0005**	1.185 (0.966–1.453)
**PSP-D**	**1.438 (1.222–1.691)**	**0.0005**	**1.237 (1.042–1.469)**
**SPON1**	**1.921 (1.432–2.577)**	**0.0005**	1.135 (0.809–1.591)
VEGFD	**1.821 (1.364–2.431)**	**0.0016**	1.146 (0.850–1.545)
TNC	**1.440 (1.196–1.735)**	**0.0037**	1.145 (0.933–1.406)
AOC3	**1.712 (1.283–2.284)**	**0.0064**	1.178 (0.863–1.609)
IGFBP-7	**1.540 (1.220–1.943)**	**0.0064**	1.029 (0.791–1.339)
IL-1RT1	**1.786 (1.310–2.436)**	**0.0064**	1.253 (0.897–1.750)
TFF3	**1.519 (1.211–1.906)**	**0.0064**	1.170 (0.917–1.493)
TIMP1	**1.602 (1.236–2.077)**	**0.0073**	1.114 (0.839–1.480)
U-PAR	**1.529 (1.200–1.949)**	**0.0109**	1.111 (0.854–1.446)
ST2	**1.350 (1.136–1.603)**	**0.0111**	1.089 (0.902–1.315)
TR	**1.349 (1.132–1.607)**	**0.0132**	1.105 (0.916–1.332)
OPG	**1.653 (1.227–2.226)**	**0.0143**	1.111 (0.802–1.539)
TFPI	**1.658 (1.222–2.251)**	**0.0171**	**1.383 (1.007–1.898)**
FCGR2A	**1.406 (1.143–1.730)**	**0.0177**	**1.288 (1.041–1.594)**
COL18A1	**1.533 (1.175–2.000)**	**0.0217**	1.155 (0.871–1.530)
CXCL1	**1.207 (1.071–1.360)**	**0.0247**	**1.159 (1.025–1.310)**
IGLC2	**1.431 (1.134–1.805)**	**0.0302**	1.204 (0.945–1.534)
IGFBP-2	**1.313 (1.098–1.570)**	**0.0332**	0.900 (0.725–1.117)
IL6	**1.168 (1.054–1.295)**	**0.0332**	1.097 (0.990–1.216)
TGM2	**1.223 (1.070–1.397)**	**0.0332**	**1.150 (1.003–1.318)**
ACE2	**1.308 (1.087–1.575)**	**0.0391**	1.045 (0.861–1.269)
CCL18	**1.280 (1.080–1.516)**	**0.0391**	**1.266 (1.063–1.508)**
CD93	**1.574 (1.154–2.148)**	**0.0391**	0.923 (0.648–1.315)
IL1RL2	**0.671 (0.509–0.884)**	**0.0391**	**0.674 (0.506–0.897)**
MMP-2	**1.475 (1.129–1.926)**	**0.0391**	0.732 (0.522–1.028)
PGLYRP1	**1.334 (1.096–1.623)**	**0.0391**	1.222 (0.997–1.496)
CCL14	**1.463 (1.123–1.906)**	**0.0391**	1.078 (0.814–1.430)
Notch 3	**1.457 (1.121–1.893)**	**0.0391**	0.863 (0.635–1.172)
OSMR	**2.187 (1.267–3.775)**	**0.0391**	1.124 (0.622–2.032)
CCL24	**1.217 (1.059–1.399)**	**0.0408**	**1.174 (1.016–1.355)**
TIMP4	**1.416 (1.108–1.808)**	**0.0408**	1.080 (0.832–1.402)
TRAIL-R2	**1.404 (1.104–1.785)**	**0.0408**	1.047 (0.821–1.336)
UMOD	**0.525 (0.333–0.830)**	**0.0408**	**0.573 (0.357–0.919)**
CD163	**1.413 (1.097–1.820)**	**0.0458**	1.153 (0.886–1.502)
ICAM1	**1.515 (1.119–2.052)**	**0.0458**	1.229 (0.897–1.684)
IL-27	**1.473 (1.110–1.956)**	**0.0458**	1.088 (0.816–1.452)
OPN	**1.286 (1.070–1.547)**	**0.0458**	0.935 (0.757–1.155)
RARRES2	**1.680 (1.154–2.446)**	**0.0458**	1.458 (0.990–2.148)
SERPINA5	**0.708 (0.551–0.910)**	**0.0458**	0.951 (0.726–1.247)
LTBP2	**1.468 (1.105–1.949)**	**0.0483**	0.831 (0.595–1.161)
CRTAC1	**0.756 (0.613–0.932)**	**0.0498**	**0.780 (0.627–0.970)**
GH	**1.103 (1.025–1.188)**	**0.0498**	1.020 (0.942–1.104)
IGFBP-1	**1.146 (1.035–1.270)**	**0.0498**	1.003 (0.896–1.122)

Significant values are given in bold.

^a^Clinical adjustment for sex, age, study drug and significant factors from Table 1 (eGFR, LVEF, NYHA class, MI, diabetes, MRA and digoxin).

A similar pattern of association was observed when using sIPTW (supplementary Table 3).

No significant interactions with individual clinical events (MI/stroke, SCD or HF hospitalization) were identified (all interaction FDR > 0.20, supplementary Table 4). Results remained unchanged when considering sIPTW analyses (supplementary Table 5).

No significant interactions were identified between the time to event and the prognostic value of biomarkers (all interaction p-values > 0.10, data not shown).

### Prediction of clinical events using a multi-marker approach

**Figure 2:**
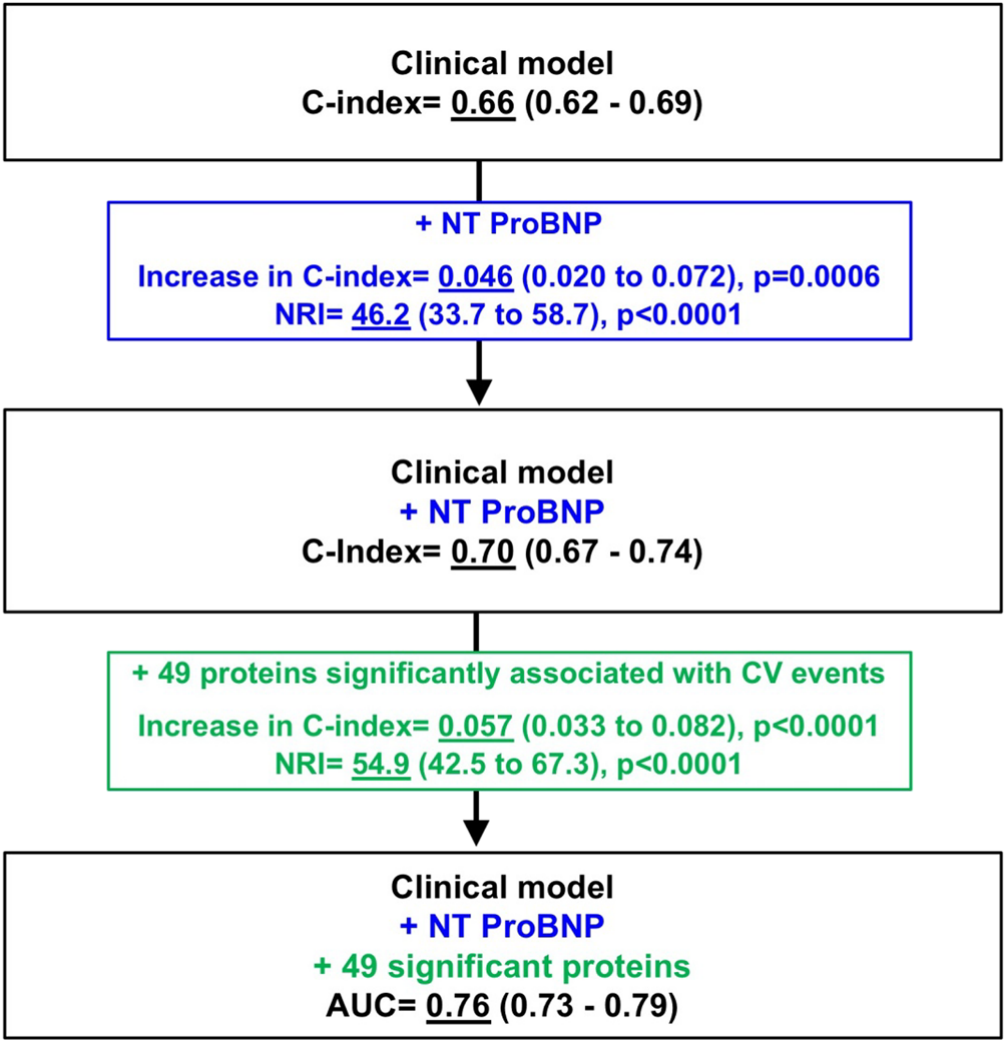
Changes in the prediction of CV events using clinical and biomarker variables. The clinical variables included in the baseline model were the following: age, sex, study treatment, eGFR, LVEF, NYHA class, history of MI, history of diabetes, MRA and digoxin.

The C-index for the clinical prediction of the composite CV events when using clinical variables was 0.66 (0.62–0.69), p < 0.0001 (Fig. 2). NT-proBNP significantly increased the C-index and provided a significant NRI increase of 46.2 (33.7 to 58.7), p < 0.0001 (Fig. 2). When further adding the biomarkers significantly associated with the aforementioned outcome, the C-index further rose to 0.760 (0.730–0.790), p < 0.0001, resulting in a C-index increase related to these biomarkers of 0.057 (0.033–0.082), p < 0.0001 and a NRI of 54.9 (42.5 to 67.3), p < 0.0001 (Fig. 2).

### Complex network analysis

**Figure 3:**
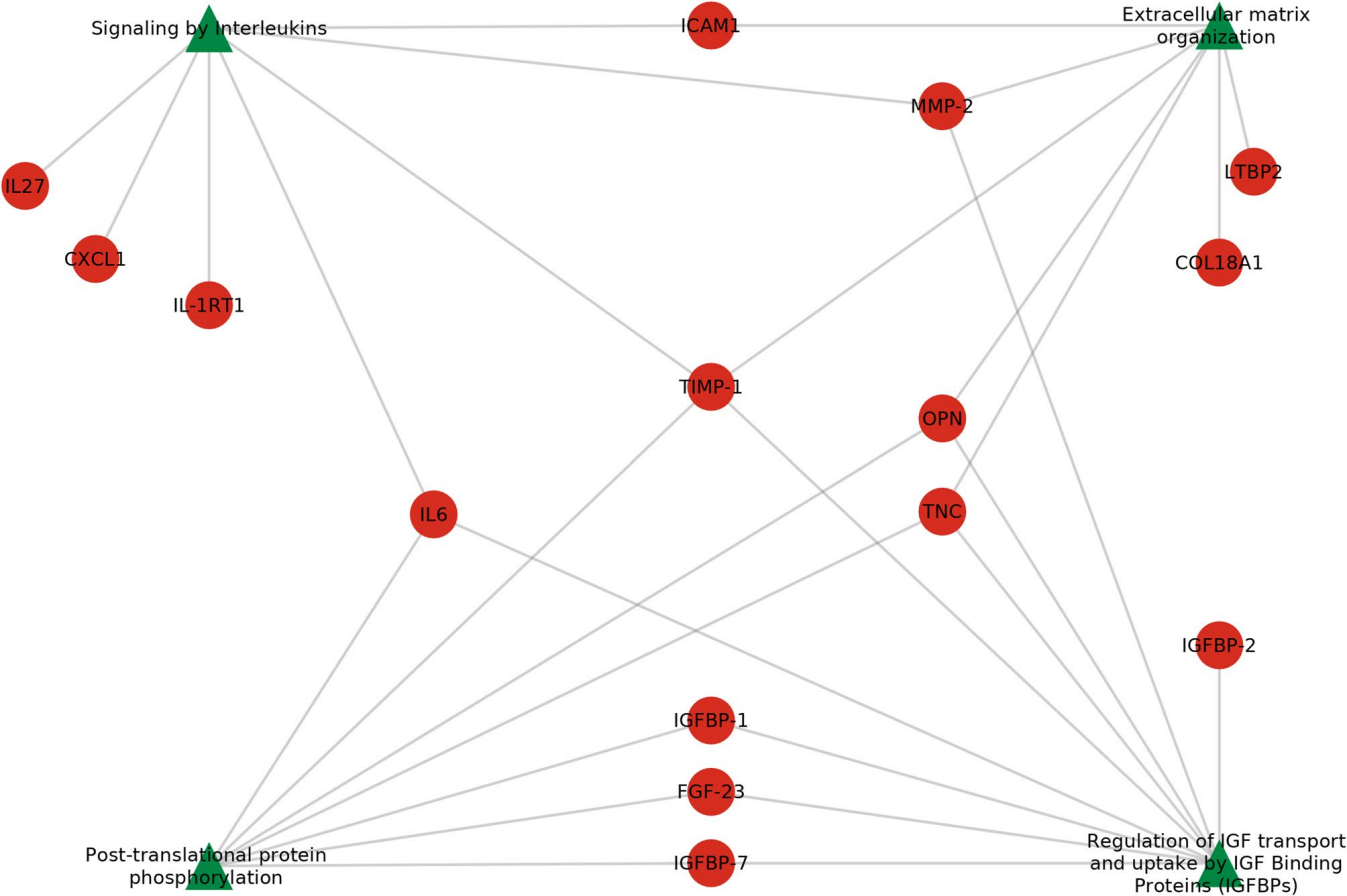
Overrepresented pathways (green triangles) linked to their significant protein biomarkers (red circles). IGFBP-2: Insulin-like growth factor-binding protein 2; IGFBP-1: Insulin-like growth factor-binding protein 1; IGFBP-7: Insulin-like growth factor-binding protein 7; FGF-23: Fibroblast growth factor 23; IL27: Interleukin-27 subunit alpha; IL-1RT1: Interleukin-1 receptor type 1; CXCL1: Growth-regulated alpha protein; IL6: Interleukin-6; LTBP2: Latent-transforming growth factor beta-binding protein 2; ICAM1: Intercellular adhesion molecule 1; TNC: Tenascin; OPN: Osteopontin; TIMP-1: Metalloproteinase inhibitor 1; MMP-2: Matrix metalloproteinase-2; COL18A1: Collagen alpha-1(XVIII) chain.

The 49 biomarker proteins associated with the sentinel events highlighted 4 overrepresented pathways (Fig. 3): “post-translational protein phosphorylation”, “regulation of IGF transport and uptake by IGF binding proteins”, “signaling by interleukins” and “extracellular matrix organization”. “Regulation of IGF transport and uptake by IGF binding proteins” was the pathway associated with the highest number of proteins (N = 9) while the 3 other pathways were each associated with 7 proteins. These pathways were linked to 15 significant biomarkers among which six were associated with one pathway and four were associated with two pathways. Four proteins (Matrix metalloproteinase-2 [MMP-2], Interleukin 6 [IL6], Osteopontin [OPN] and Tenascin [TNC]) were related to three pathways. Metalloproteinase inhibitor 1 (TIMP-1) was the only protein associated with all of the pathways.

## Discussion

We identified 49 proteins significantly associated with clinical CV events in patients with HFrEF and underlying CAD following a worsening HF episode. Unsurprisingly, natriuretic peptides were among the best predictors of subsequent clinical CV events, along with TIMD4, FGF-23, GDF-15, PSP-D and SPON1, biomarkers associated with “post-translational protein phosphorylation”, “regulation of IGF transport and uptake by IGF binding proteins”, “signaling by interleukins” and “extracellular matrix organization” in network analysis, and in which TIMP-1 appears as a central node. While identification of these pathways may indicate that they play a role in the subsequent clinical events that were experienced by patients in the study, we could not identify a significant interaction between biomarkers and the clinical event per se (i.e. either MI/stroke, SCD or HF hospitalization) in these patients.

### Protein biomarkers are homogenously associated with different outcomes

Our analysis intended to identify different biological profiles underlying distinct CV clinical events. Yet, none of the biomarkers identified herein were differentially associated with MI/stroke, SCD or HF hospitalization, respectively, when assessing formal statistical interactions while accounting for multiplicity of tests (all p values for interaction > 0.20). This result is consistent with the possibility that there are unifying underling mechanisms to all clinical events in a population that is comprised of patients with CAD and HFrEF who are admitted for worsening HF. The pathways related to the biomarkers identified were “post-translational protein phosphorylation”, “regulation of IGF transport and uptake by IGF binding proteins”, “signaling by interleukins” and “extracellular matrix organization”. These pathways relate to the broad concept of inflammation and remodeling, both of which are central to HF development and worsening but are also very much associated with stroke and MI biology. Our results could also suggest that there is some overlap within the pathways involved in these events. For instance, MI is a significant cause of HF initiation and worsening. In addition, stroke is very much associated with MI, and both MI and stroke are frequently associated with CV death in patients with HF. Differentiating distinct biological profiles for these events may hence be challenging due to the strong mechanistic and prognostic interplay of these clinical events with one another.

### The value of natriuretic peptides to predict a range of CV outcomes in patients with HFrEF admitted for worsening HF

Our study provides proteomic results for a large population of patients recently hospitalized for HF, a population that has been less well studied comparatively to ambulatory HF. Natriuretic peptides were unsurprisingly among the best predictors of subsequent clinical events in the specific context of HF hospitalization. However, our analyses emphasize that natriuretic peptides also predict a range of CV outcomes, including ischemic events (MI/stroke) and SCD, suggesting that they may be a pleiotropic/omnipotent predictor of events in patients with HFrEF following HF hospitalization. Thus, elevated NT-proBNP should not be perceived solely as a risk for HF related outcomes, but rather as an indicator of overall risk of CV events^16^ (along with LVEF^17^ and eGFR, as emphasized in Table 2).

### TIMD4, FGF-23, GDF-15, PSP-D and SPON1 as key predictors of outcome in patients with HFrEF admitted for worsening HF

The next best predictors of events in our study were TIMD4, FGF-23, GDF-15, PSP-D and SPON1. Importantly, none of these 5 proteins were highlighted in the enriched network of upregulated proteins in ischemic HF relative to non-ischemic HF in the BIOSTAT cohort^18^. This striking difference may suggest that proteins related to the HF etiology might not be related to prognosis in patients with ischemic HF.

There is only sparse evidence regarding TIMD4 in CV disease. TIMD4 haplotypes have been reported to be associated with CAD and stroke as well as statin therapy in a case–control study performed in Chinese populations^19^. In our analysis, despite the non-significant statistical interaction, it should be acknowledged that the point estimate of associations were numerically higher for MI/stroke (OR = 2.832, (1.701–4.713), p = 0.0086) than for the other CV outcomes (OR for SCD = 1.853, (1.240–2.768), p = 0.0769; OR for HFH = 1.304, (0.928–1.831), p = 0.8652; supplementary Table 3).

FGF-23 has been shown to predict death and/or HFH in both ambulatory and hospitalized patients^4,20^. Of note, in the BIOSTAT cohort, in which both ambulatory and hospitalized patients were included, FGF-23 was similarly retained in multivariable models in inpatients (HR = 1.13 (1.02, 1.24)) and outpatients (HR = 1.32 (1.14, 1.53)) with HF^4^. We further show herein that, in patients hospitalized for HF in the context of CAD, FGF-23 is also a key predictor of CV outcome (even when considering non-HF outcomes). Taken together, these data suggest that FGF-23, alongside with NT-proBNP, is a potent biomarker for overall CV risk prediction in HF.

GDF-15 is associated with death and/or HFH in hospitalized^4,21^ patients with HF. This biomarker has also been shown to decrease during hospital stay in concomitance with improvement of signs and symptoms^21^, which suggests that it may be partially related to congestion. In the BIOSTAT cohort, GDF-15 was one of the main biomarkers differentiating inpatient and outpatient settings, with GDF-15 being higher in inpatients^4^. However, these 2 previous cohorts reported contrasting associations: Lourenço et al. reported that, in multivariable analysis, patients with either elevated admission or discharge GDF-15 had a significant twofold increase in 1-year death risk^21^. In contrast, in the BIOSTAT cohort, higher GDF-15 levels were associated with lower rates of HF hospitalization in the inpatient subgroup (HR = 0.80 (0.67, 0.95), p = 0.01)^4^, whereas GDF-15 was not retained as a significant predictor in the outpatient subgroup. Our results support the findings of Lourenço et al. and suggest that GDF-15 predicts a range of CV events such as MI/stroke and SCD (OR = 1.736, (1.265–2.380), p = 0.0345; supplementary Table 3) in the setting of hospitalized HF with CAD history.

Limited evidence exists regarding PSP-D in HF. Surfactant-derived proteins have been emphasized as markers of alveolar membrane damage in HF^22^. In addition, PSP-D trajectories over time have also been shown to be associated with the composite outcome of HFH, cardiac death, LVAD-placement and heart transplantation in a cohort of chronic HF patients^23^. Our results further suggest that PSP-D is important in more acute settings, which is in line with the previously reported involvement of PSP-D in alveolar damage^22^, an event likely to occur in worsening HF.

SPON-1 has been reported to be associated with the risk of HF hospitalization in chronic HF with CKD^24^. Increase in plasma levels of SPON1 has also been shown to precede adverse HF-related events and CV death in patients with CHF^25^ in a serial plasma measurements study using joint modeling. These prior data are in keeping with our results showing the strong prognostic value of SPON-1 observed in hospitalized patients with often some level of concomitant worsening in renal function.

Importantly, all of these proteins, along with the other proteins significantly associated with outcome as highlighted in the network analysis, relate to the broad concept of inflammation and remodeling.

### TIMP-1 as a central node in pathways associated with higher risk in worsening HF with CAD

In our network analysis, TIMP-1 was associated with all pathways identified as overrepresented from the 49 biomarker proteins (Fig. 2). This factor is known to be associated with “post-translational protein phosphorylation”, “regulation of IGF transport and uptake by IGF binding proteins”, “signaling by interleukins” and “extracellular matrix organization”. Importantly, TIMP-1 regulates various metalloprotease activity^26^ including MMP and a disintegrin as well as metalloproteinases (ADAMs). TIMP-1 is consequently closely related to cardiovascular remodeling^26^. It has specifically been reported to modulate inflammation and extracellular matrix fibrosis following injury^27^, an aspect that could be particularly relevant in the setting of acute HF and/or ischemic HF. Importantly, this biomarker was not measured in previous studies assessing proteomics in HF (such as the BIOSTAT cohort^4^). Polymorphisms in the *TIMP-1* (and *TIMP-2*) genes have been shown to be associated with poor outcome in chronic HF^28^. TIMP-1 is elevated in HF patients when compared to healthy populations^29^, and higher blood levels of TIMP-1 are negatively correlated with peak VO2 in HF patients^29^. A recent meta-analysis of animal models showed that TIMP-1 is consistently induced in hemodynamic models, and is associated with cardiac fibrosis^30^. The central place of TIMP-1 in the current complex network is in line with the central and unifying place of fibrosis in progressive HF deterioration.

## Limitations

Certain limitations should be pointed out in the present study.

First, the case–control study was designed to identify protein biomarkers for CV risk, although cannot provide any information regarding causality. This case–control design was chosen because of logistical/cost constrains; a cohort design would have decreased the risk of bias.

In addition, the proteomics assay did not provide standard concentration units, making comparisons with clinically applied cutoffs difficult. Still, the Olink standard procedures ensure a good correlation with standard measurement methodologies, and in this mechanistic screening study, there was no intention to measure specific concentrations of biomarkers, but only to investigate the overexpression of proteins associated with target mechanistic pathways.

Pre-selection is an inherent limitation of our proteomic analysis. Indeed, the choice of panels was based on proteins that have previously been associated with CV disease. Therefore an opportunity to discover additional new proteins not covered by the selected CVD panels and of potential prognostic importance could have possibly been missed. Similarly, our approach was focused on proteins, thus potentially missing integrated clinical/biological phenotypes relevant to the prediction of specific CV outcomes.

Interaction analyses are known to lack statistical power. Consequently, our analysis may have missed “true” differential associations of biomarkers with specific outcomes given the relatively modest sample size of the study. However, genuinely strong determinants of distinct types of events are unlikely to have been missed.

Our analysis was limited by its case–control nature and the central place of TIMP1 should be further assessed in cohort studies.

The inclusion of patients could occur up to several weeks after patient admission for worsening HF. This does introduce some variability across measurements and may have some impact on the preciseness of the associations with the outcomes identified herein.

Importantly, this study comprised patients with HFrEF. Given the differences in biological phenotypes observed in HF according to different levels of LVEF^31^, these results likely do not apply to patients with HF with preserved LVEF.

Due to the moderate sample size and the matched nature of the dataset, we were unable to provide an additional interaction analysis with patient characteristics, e.g. LVEF.

Our pathway analysis is also limited by the number of proteins identified as associated with outcome. Additional biological analyses including RNA sequencing tools could help in better characterizing involved biological pathways.

A replication phase involving a prospective validation of these biomarkers in other populations is required to improve the external validation of these results. Finally, the implementation of these results in the future would likely necessitate a broader access to proteomic data and web-based software able to provide a risk calculation based on proteomic data. Numerous steps are still needed to implement these approaches in clinical practice.

Lastly, only CV outcomes were considered in this analysis. Consideration to non-CV events (e.g. cancer) might have helped in identifying CV-specific biological phenotypes (even if not specific to a particular CV event). In addition, patients with a given event could experience another event during follow-up (i.e. there was some overlap between events) which may have hampered our ability to identify specific profiles of biomarkers related to a given clinical event. Nevertheless, such event overlap is actually what patients experience in routine practice, and this overlap is consequently inherent to cardiovascular medicine.

## Conclusion

We identified 49 proteins significantly associated with clinical events in patients with HFrEF and underlying CAD following a worsening HF episode. In this setting, the added value of these biomarkers for risk prediction was of similar magnitude to that derived from natriuretic peptides. Natriuretic peptides however appear as pleiotropic and potent predictors of events as they are similarly associated with HF-related events, ischemic (MI/stroke) events and SCD. In addition, TIMD4, FGF-23, GDF-15, PSP-D and SPON1 appear as key predictors of outcome in patients with HFrEF admitted for worsening HF, and position TIMP-1 as a central pathophysiological node in this setting.

## Supplementary Information

Supplementary Tables.
