# Supplementary material for: Inflammation and remodeling pathways and risk of cardiovascular events in patients with ischemic heart failure and reduced ejection fraction
Source: Sci Rep. 2022 May 20;12:8574. doi: 10.1038/s41598-022-12385-0 (PMC9123183; doi:10.1038/s41598-022-12385-0)
Supplement: Supplementary file 1 — Supplementary Tables. [file 41598_2022_12385_MOESM1_ESM.docx]

**Supplementary Table 1: Overlap of events in cases considered in the analysis**

| **Patients with MI** |  |
| --- | --- |
| No other event | 30 (34.5%) |
| One additional event |  |
| With SCD | 31 (35.6%) |
| With HFH | 14 (16.1%) |
| Two additional events |  |
| With HFH and SCD | 8 (9.2%) |
| With stroke and SCD | 3 (3.5%) |
| Three additional events |  |
| With stroke, HFH and SCD | 1 (1.1%) |
| **Patients with stroke** |  |
| No other event | 27 (60.0%) |
| One additional event |  |
| With SCD | 14 (31.1%) |
| With HFH | 1 (2.2%) |
| With MI | 1 (2.2%) |
| Two additional events |  |
| With HFH and SCD | 1 (2.2%) |
| With MI and SCD | 1 (2.2%) |
| **Patients with SCD** |  |
| No other event | 152 (99.4%) |
| One additional event |  |
| With stroke | 1 (0.6%) |
| **Patients with HFH** |  |
| No other event | 128 (64.0%) |
| One additional event |  |
| With SCD | 62 (31.0%) |
| With stroke | 4 (2.0%) |
| With MI | 1 (0.5%) |
| Two additional events |  |
| With MI and SCD | 3 (1.5%) |
| With stroke and SCD | 2 (1.0%) |

**Supplementary Table 2: Patient characteristics according to MI/stroke, SCD, HF rehospitalization overall in the sex and age-matched COMMANDER HF population**

|  |  | **MI/ Stroke** | | | | **SCD** | | | **HF Rehospitalization** | | | | |
| --- | --- | --- | --- | --- | --- | --- | --- | --- | --- | --- | --- | --- | --- |
|  |  | **Controls** | **Cases** | **SMD** | **P** | **Controls** | **Cases** | **SMD** | **P** | **Controls** | **Cases** | **SMD** | **P** |
| **Characteristic** |  | **(n=127)** | **(n=132)** |  |  | **(n=149)** | **(n=153)** |  |  | **(n=179)** | **(n=200)** |  |  |
| Age (yrs) |  | 68.1 ± 10.4 | 67.7 ± 10.6 | 4.40% | 0.72 | 66.6 ± 10.1 | 66.7 ± 10.0 | 1.10% | 0.92 | 67.6 ± 9.7 | 67.5 ± 10.2 | 1.40% | 0.89 |
| Study drug |  | 60 (47.2%) | 56 (42.1%) | 10.40% | 0.45 | 68 (45.6%) | 73 (47.7%) | 4.20% | 0.73 | 82 (45.8%) | 101 (50.5%) | 9.40% | 0.41 |
| Female sex |  | 38 (29.9%) | 41 (30.8%) | 2.00% | 0.89 | 29 (19.5%) | 31 (20.3%) | 2.00% | 0.89 | 43 (24.0%) | 48 (24.0%) | 0.10% | 1 |
| Race |  |  |  | 24.90% | 0.54 |  |  | 16.90% | 0.43 |  |  | 15.80% | 0.66 |
| White |  | 114 (89.8%) | 120 (90.2%) |  |  | 133 (89.3%) | 143 (93.5%) |  |  | 158 (88.3%) | 170 (85.0%) |  |  |
| Black |  | 0 (0.0%) | 2 (1.5%) |  |  | 2 (1.3%) | 1 (0.7%) |  |  | 0 (0.0%) | 2 (1.0%) |  |  |
| Asian |  | 12 (9.4%) | 11 (8.3%) |  |  | 12 (8.1%) | 6 (3.9%) |  |  | 18 (10.1%) | 24 (12.0%) |  |  |
| Other |  | 1 (0.8%) | 0 (0.0%) |  |  | 2 (1.3%) | 3 (2.0%) |  |  | 3 (1.7%) | 4 (2.0%) |  |  |
| Region |  |  |  | 17.30% | 0.68 |  |  | 34.00% | 0.058 |  |  | 33.60% | 0.042 |
| Eastern Europe |  | 85 (66.9%) | 88 (66.2%) |  |  | 102 (68.5%) | 123 (80.4%) |  |  | 128 (71.5%) | 121 (60.5%) |  |  |
| North America |  | 2 (1.6%) | 1 (0.8%) |  |  | 3 (2.0%) | 2 (1.3%) |  |  | 0 (0.0%) | 6 (3.0%) |  |  |
| Asia Pacific |  | 12 (9.4%) | 11 (8.3%) |  |  | 12 (8.1%) | 6 (3.9%) |  |  | 18 (10.1%) | 24 (12.0%) |  |  |
| Latin America |  | 13 (10.2%) | 10 (7.5%) |  |  | 22 (14.8%) | 10 (6.5%) |  |  | 15 (8.4%) | 18 (9.0%) |  |  |
| Western Europe |  | 15 (11.8%) | 23 (17.3%) |  |  | 10 (6.7%) | 12 (7.8%) |  |  | 18 (10.1%) | 31 (15.5%) |  |  |
| BMI (kg/m2) |  | 27.4 ± 5.1 | 27.9 ± 5.2 | 9.00% | 0.47 | 27.6 ± 4.8 | 27.6 ± 5.0 | 0.40% | 0.97 | 27.7 ± 5.1 | 28.2 ± 5.2 | 9.20% | 0.37 |
| **eGFR (ml/min/1.73 m2)** |  | **66.8 ± 21.7** | **65.0 ± 24.1** | **7.60%** | **0.54** | **72.7 ± 23.3** | **66.3 ± 22.3** | **28.10%** | **0.016** | **70.4 ± 23.2** | **63.2 ± 22.7** | **31.40%** | **0** |
| **eGFR (ml/min/1.73 m2)** |  |  |  | **18.40%** | **0.67** |  |  | **32.00%** | **0.036** |  |  | **27.40%** | **0.07** |
| <30 ml/min/1.73 m2 |  | 4 (3.1%) | 8 (6.0%) |  |  | 2 (1.3%) | 3 (2.0%) |  |  | 5 (2.8%) | 14 (7.0%) |  |  |
| 30 to <60 ml/min/1.73 m2 |  | 55 (43.3%) | 56 (42.1%) |  |  | 41 (27.5%) | 65 (42.5%) |  |  | 61 (34.1%) | 78 (39.0%) |  |  |
| 60 to <90 ml/min/1.73 m2 |  | 50 (39.4%) | 54 (40.6%) |  |  | 73 (49.0%) | 61 (39.9%) |  |  | 81 (45.3%) | 86 (43.0%) |  |  |
| >=90 ml/min/1.73 m2 |  | 18 (14.2%) | 15 (11.3%) |  |  | 33 (22.1%) | 24 (15.7%) |  |  | 32 (17.9%) | 22 (11.0%) |  |  |
| BNP level (pg/ml) |  | 924.7 ± 839.0 | 860.8 ± 557.3 | 8.70% | 0.79 | 856.9 ± 720.3 | 1045.6 ± 861.6 | 24.10% | 0.38 | 859.4 ± 831.9 | 929.3 ± 714.1 | 9.00% | 0.7 |
| Log2 BNP (pg/ml) |  |  |  |  |  |  |  |  |  |  |  |  |  |
| BNP rank (pg/ml) |  | 19.2 ± 12.8 | 21.2 ± 9.3 | 18.00% | 0.57 | 26.1 ± 16.3 | 30.6 ± 15.6 | 28.00% | 0.3 | 33.5 ± 21.4 | 38.4 ± 19.9 | 23.90% | 0.31 |
| NT-proBNP (pg/ml) |  | 5966 ± 11346 | 4396 ± 4464 | 17.80% | 0.31 | 5209 ± 7171 | 6835 ± 10423 | 18.10% | 0.25 | 6312 ± 7619 | 5242 ± 5229 | 16.40% | 0.22 |
| Log2 NT-proBNP (pg/ml) |  |  |  |  |  | 8.0 ± 1.0 | 8.2 ± 1.0 | 22.20% | 0.14 |  |  |  |  |
| NT-proBNP rank |  | 74.6 ± 41.2 | 72.3 ± 43.8 | 5.50% | 0.74 |  |  |  |  | 109.4 ± 70.0 | 114.5 ± 58.9 | 7.90% | 0.55 |
| D-dimer level (mg/L) |  | 545.6 ± 614.7 | 801.6 ± 1456.0 | 22.70% | 0.093 | 634.7 ± 1002.2 | 698.5 ± 742.3 | 7.30% | 0.53 | 704.4 ± 1225.3 | 712.2 ± 860.7 | 0.70% | 0.94 |
| Log2 D-dimer |  | 5.9 ± 0.8 | 6.2 ± 0.9 | 30.90% | 0.015 | 6.0 ± 0.9 | 6.2 ± 0.8 | 20.60% | 0.075 |  |  |  |  |
| D-dimer rank |  |  |  |  |  |  |  |  |  | 172.2 ± 116.6 | 205.9 ± 100.4 | 31.10% | 0.003 |
| **Ejection fraction (%)** |  | **33.5 ± 6.5** | **32.6 ± 6.5** | **14.10%** | **0.26** | **33.2 ± 6.7** | **30.7 ± 7.2** | **36.10%** | **0.002** | **32.6 ± 6.7** | **30.4 ± 7.7** | **31.10%** | **0** |
| **Ejection fraction rank** |  | **137.5 ± 75.8** | **123.8 ± 73.8** | **18.20%** | **0.14** | **168.2 ± 85.8** | **135.3 ± 85.6** | **38.40%** | **0.001** | **206.8 ± 106.4** | **174.9 ± 109.9** | **29.50%** | **0.01** |
| NYHA |  |  |  | 20.30% | 0.99 |  |  | 29.00% | 0.068 |  |  | 18.10% | 0.18 |
| I |  | 2 (1.6%) | 3 (2.3%) |  |  | 5 (3.4%) | 2 (1.3%) |  |  | 6 (3.4%) | 8 (4.0%) |  |  |
| II |  | 63 (49.6%) | 67 (50.4%) |  |  | 72 (48.3%) | 58 (37.9%) |  |  | 90 (50.3%) | 88 (44.0%) |  |  |
| III |  | 61 (48.0%) | 61 (45.9%) |  |  | 71 (47.7%) | 88 (57.5%) |  |  | 82 (45.8%) | 97 (48.5%) |  |  |
| IV |  | 1 (0.8%) | 2 (1.5%) |  |  | 1 (0.7%) | 5 (3.3%) |  |  | 1 (0.6%) | 7 (3.5%) |  |  |
| **Myocardial infarction** |  | **95 (74.8%)** | **108 (81.2%)** | **15.50%** | **0.23** | **107 (71.8%)** | **123 (80.4%)** | **20.20%** | **0.1** | **129 (72.1%)** | **154 (77.0%)** | **11.30%** | **0.29** |
| Stroke |  | 17 (13.4%) | 14 (10.5%) | 8.80% | 0.57 | 9 (6.0%) | 16 (10.5%) | 16.10% | 0.21 | 18 (10.1%) | 28 (14.0%) | 12.10% | 0.27 |
| **Diabetes** |  | **43 (33.9%)** | **69 (51.9%)** | **37.00%** | **0.004** | **57 (38.3%)** | **67 (43.8%)** | **11.30%** | **0.35** | **63 (35.2%)** | **87 (43.5%)** | **17.10%** | **0.11** |
| Hypertension |  | 96 (75.6%) | 106 (79.7%) | 9.90% | 0.46 | 112 (75.2%) | 117 (76.5%) | 3.00% | 0.89 | 131 (73.2%) | 152 (76.0%) | 6.50% | 0.56 |
| ACEI or ARB |  | 118 (92.9%) | 127 (95.5%) | 11.00% | 0.43 | 144 (96.6%) | 145 (94.8%) | 9.30% | 0.57 | 169 (94.4%) | 180 (90.0%) | 16.50% | 0.13 |
| Beta blockers |  | 119 (93.7%) | 121 (91.0%) | 10.30% | 0.49 | 139 (93.3%) | 140 (91.5%) | 6.70% | 0.67 | 166 (92.7%) | 184 (92.0%) | 2.80% | 0.85 |
| **MRA** |  | **92 (72.4%)** | **101 (75.9%)** | **8.00%** | **0.57** | **104 (69.8%)** | **131 (85.6%)** | **38.70%** | **0.001** | **131 (73.2%)** | **151 (75.5%)** | **5.30%** | **0.64** |
| **Digoxin** |  | **5 (3.9%)** | **10 (7.5%)** | **15.50%** | **0.29** | **9 (6.0%)** | **19 (12.4%)** | **22.20%** | **0.073** | **6 (3.4%)** | **14 (7.0%)** | **16.50%** | **0.17** |
| Aspirin |  | 115 (90.6%) | 124 (93.2%) | 9.80% | 0.5 | 143 (96.0%) | 147 (96.1%) | 0.50% | 1 | 168 (93.9%) | 179 (89.5%) | 15.80% | 0.14 |

**Supplementary Table 3*:* sIPTW analysis of significant associations of protein biomarkers with CV events (as listed in Table 2).**

| **Biomarker** | **sIPTW logistic model** | | **sIPTW logistic model**  **adjusted for NT-proBNP** |
| --- | --- | --- | --- |
|  | **OR (95% CI)** | **FDR** | **OR (95% CI)** |
| **NT-proBNP** | **1.302 (1.205 - 1.406)** | **<.0001** |  |
| **BNP** | **1.258 (1.178 - 1.343)** | **<.0001** |  |
| **TIMD4** | **1.719 (1.378 - 2.144)** | **0.0001** | **1.502 (1.196 - 1.886)** |
| **FGF-23** | **1.229 (1.121 - 1.348)** | **0.0006** | 1.068 (0.964 - 1.185) |
| **GDF-15** | **1.391 (1.186 - 1.631)** | **0.0015** | 1.086 (0.905 - 1.303) |
| **PSP-D** | **1.421 (1.219 - 1.655)** | **0.0004** | 1.247 (1.062 - 1.464) |
| **SPON1** | **1.895 (1.435 - 2.502)** | **0.0004** | 1.156 (0.833 - 1.605) |
| VEGFD | **1.743 (1.338 - 2.271)** | **0.0015** | 1.157 (0.877 - 1.527) |
| TNC | **1.465 (1.220 - 1.759)** | **0.0015** | 1.194 (0.980 - 1.456) |
| AOC3 | **1.656 (1.267 - 2.165)** | **0.0056** | 1.165 (0.870 - 1.561) |
| IGFBP-7 | **1.465 (1.186 - 1.810)** | **0.0078** | 0.999 (0.782 - 1.277) |
| IL-1RT1 | **1.715 (1.291 - 2.280)** | **0.0056** | 1.205 (0.883 - 1.645) |
| TFF3 | **1.350 (1.122 - 1.623)** | **0.0202** | 1.023 (0.833 - 1.258) |
| TIMP1 | **1.576 (1.233 - 2.013)** | **0.0064** | 1.090 (0.828 - 1.436) |
| U-PAR | **1.456 (1.170 - 1.811)** | **0.0121** | 1.045 (0.818 - 1.335) |
| ST2 | **1.360 (1.151 - 1.607)** | **0.0064** | 1.117 (0.932 - 1.339) |
| TR | **1.340 (1.135 - 1.582)** | **0.0101** | 1.111 (0.930 - 1.328) |
| OPG | **1.591 (1.211 - 2.090)** | **0.0129** | 1.086 (0.803 - 1.469) |
| TFPI | **1.663 (1.244 - 2.225)** | **0.0104** | 1.457 (1.079 - 1.968) |
| FCGR2A | **1.400 (1.147 - 1.708)** | **0.0135** | 1.257 (1.025 - 1.542) |
| COL18A1 | **1.398 (1.109 - 1.763)** | **0.0372** | 1.027 (0.798 - 1.323) |
| CXCL1 | **1.189 (1.063 - 1.331)** | **0.0308** | 1.152 (1.026 - 1.293) |
| IGLC2 | **1.359 (1.101 - 1.678)** | **0.0355** | 1.117 (0.894 - 1.396) |
| IGFBP-2 | **1.260 (1.076 - 1.475)** | **0.0355** | 0.869 (0.713 - 1.060) |
| IL6 | **1.175 (1.063 - 1.299)** | **0.0211** | 1.096 (0.991 - 1.212) |
| TGM2 | **1.207 (1.066 - 1.368)** | **0.0322** | 1.138 (1.001 - 1.293) |
| ACE2 | **1.281 (1.083 - 1.515)** | **0.0355** | 1.039 (0.870 - 1.242) |
| CCL18 | **1.283 (1.092 - 1.507)** | **0.0303** | 1.232 (1.044 - 1.454) |
| CD93 | **1.471 (1.115 - 1.940)** | **0.043** | 0.867 (0.626 - 1.201) |
| IL1RL2 | **0.699 (0.541 - 0.904)** | **0.043** | 0.715 (0.549 - 0.930) |
| MMP-2 | **1.445 (1.128 - 1.851)** | **0.0355** | 0.758 (0.550 - 1.044) |
| PGLYRP1 | **1.303 (1.085 - 1.566)** | **0.0372** | 1.147 (0.948 - 1.388) |
| Notch 3 | **1.433 (1.125 - 1.825)** | **0.0355** | 0.855 (0.638 - 1.145) |
| OSMR | **2.203 (1.312 - 3.699)** | **0.031** | 1.181 (0.672 - 2.076) |
| CCL24 | **1.222 (1.072 - 1.393)** | **0.031** | 1.200 (1.048 - 1.374) |
| IGFBP-1 | **1.143 (1.040 - 1.256)** | **0.042** | 0.990 (0.890 - 1.101) |
| CD163 | **1.425 (1.119 - 1.815)** | **0.0355** | 1.177 (0.914 - 1.516) |
| MMP-9 | **1.153 (1.042 - 1.276)** | **0.043** | 1.146 (1.033 - 1.272) |
| ICAM1 | **1.526 (1.144 - 2.037)** | **0.0355** | 1.274 (0.944 - 1.719) |
| OPN | **1.257 (1.062 - 1.487)** | **0.0493** | 0.942 (0.777 - 1.142) |
| SERPINA5 | **0.721 (0.570 - 0.912)** | **0.043** | 0.932 (0.725 - 1.199) |
| LTBP2 | **1.464 (1.121 - 1.911)** | **0.039** | 0.835 (0.605 - 1.152) |
| CRTAC1 | **0.768 (0.633 - 0.932)** | **0.0488** | 0.782 (0.641 - 0.955) |

**Supplementary Table 4: Associations of protein biomarkers (significantly associated with overall CV events) with specific clinical events (MI/stroke, SCD, HF rehospitalization).**

| **Biomarker** |  | **MI/Stroke** | | **SCD** | | **Rehospitalization**  **for HF** | |  | **Interaction** |
| --- | --- | --- | --- | --- | --- | --- | --- | --- | --- |
|  |  | **OR (95% CI)** | **FDR** | **OR (95% CI)** | **FDR** | **OR (95% CI)** | **FDR** |  | **FDR** |
| NT-proBNP |  | **1.343 (1.140 - 1.582)** | **0.0293** | **1.504 (1.284 - 1.761)** | **<0.0001** | **1.319 (1.162 - 1.497)** | **0.0051** |  | 0.7671 |
| BNP |  | **1.262 (1.100 - 1.447)** | **0.0411** | **1.414 (1.241 - 1.612)** | **<0.0001** | 1.233 (1.110 - 1.370) | 0.0135 |  | 0.7668 |
| TIMD4 |  | **2.832 (1.701 - 4.713)** | **0.0086** | 1.853 (1.240 - 2.768) | 0.0769 | 1.304 (0.928 - 1.831) | 0.8652 |  | 0.6075 |
| FGF-23 |  | 1.244 (1.014 - 1.527) | 0.213 | 1.289 (1.093 - 1.520) | 0.0769 | 1.258 (1.075 - 1.472) | 0.2889 |  | 0.9766 |
| GDF-15 |  | 1.425 (1.046 - 1.940) | 0.1786 | **1.736 (1.265 - 2.380)** | **0.0345** | 1.431 (1.076 - 1.904) | 0.5591 |  | 0.8528 |
| PSP-D |  | **2.111 (1.507 - 2.958)** | **0.0039** | 1.451 (1.094 - 1.923) | 0.1198 | 1.125 (0.878 - 1.442) | 0.8652 |  | 0.596 |
| SPON1 |  | 2.407 (1.394 - 4.155) | 0.0637 | 1.868 (1.109 - 3.147) | 0.1492 | 1.665 (1.065 - 2.605) | 0.6396 |  | 0.8528 |
| VEGFD |  | 2.163 (1.214 - 3.855) | 0.1475 | 2.031 (1.206 - 3.422) | 0.1198 | 1.537 (1.012 - 2.335) | 0.8107 |  | 0.8528 |
| TNC |  | 1.525 (1.073 - 2.167) | 0.1668 | 1.935 (1.339 - 2.796) | 0.0304 | 1.185 (0.902 - 1.557) | 0.8652 |  | 0.6912 |
| AOC3 |  | 1.870 (1.076 - 3.252) | 0.1876 | 1.423 (0.861 - 2.352) | 0.3692 | 1.841 (1.189 - 2.853) | 0.3453 |  | 0.8864 |
| IGFBP-7 |  | 1.667 (1.069 - 2.598) | 0.1786 | 1.798 (1.187 - 2.724) | 0.1071 | 1.308 (0.921 - 1.858) | 0.8652 |  | 0.8365 |
| IL-1RT1 |  | 1.926 (1.115 - 3.325) | 0.1668 | 1.694 (0.968 - 2.965) | 0.2345 | 1.761 (1.074 - 2.887) | 0.6396 |  | 0.9705 |
| TFF3 |  | 1.539 (1.062 - 2.230) | 0.1786 | **1.933 (1.311 - 2.851)** | **0.0407** | 1.275 (0.925 - 1.757) | 0.8652 |  | 0.7668 |
| TIMP1 |  | 2.302 (1.309 - 4.045) | 0.1299 | 1.752 (1.121 - 2.738) | 0.1358 | 1.255 (0.855 - 1.844) | 0.8652 |  | 0.74 |
| U-PAR |  | 1.796 (1.158 - 2.786) | 0.1475 | 1.702 (1.133 - 2.558) | 0.1202 | 1.250 (0.862 - 1.813) | 0.8652 |  | 0.7671 |
| ST2 |  | 1.599 (1.087 - 2.352) | 0.1663 | 1.442 (1.087 - 1.913) | 0.1224 | 1.185 (0.911 - 1.542) | 0.8652 |  | 0.7671 |
| TR |  | 1.289 (0.931 - 1.786) | 0.3107 | 1.356 (0.990 - 1.858) | 0.2185 | 1.388 (1.056 - 1.825) | 0.6396 |  | 0.9705 |
| OPG |  | 1.662 (0.989 - 2.794) | 0.2501 | 1.760 (1.031 - 3.006) | 0.1999 | 1.579 (0.994 - 2.507) | 0.8107 |  | 0.9757 |
| TFPI |  | 2.130 (1.231 - 3.687) | 0.1475 | 1.790 (1.010 - 3.172) | 0.2122 | 1.272 (0.781 - 2.069) | 0.8652 |  | 0.7671 |
| FCGR2A |  | 1.718 (1.130 - 2.611) | 0.1475 | 1.269 (0.888 - 1.812) | 0.3957 | 1.340 (0.968 - 1.853) | 0.8606 |  | 0.8492 |
| COL18A1 |  | 1.410 (0.875 - 2.270) | 0.3482 | 1.777 (1.137 - 2.776) | 0.1229 | 1.442 (0.971 - 2.143) | 0.8606 |  | 0.8867 |
| CXCL1 |  | 1.322 (1.065 - 1.641) | 0.1475 | 1.272 (1.032 - 1.570) | 0.1541 | 1.080 (0.891 - 1.309) | 0.8656 |  | 0.7671 |
| IGLC2 |  | 1.851 (1.190 - 2.879) | 0.1475 | 1.366 (0.933 - 1.999) | 0.3046 | 1.249 (0.875 - 1.782) | 0.8652 |  | 0.7671 |
| IGFBP-2 |  | 1.289 (0.942 - 1.765) | 0.3107 | 1.576 (1.170 - 2.124) | 0.0769 | 1.132 (0.858 - 1.493) | 0.8652 |  | 0.7668 |
| IL6 |  | 1.201 (0.971 - 1.485) | 0.2916 | 1.220 (1.034 - 1.440) | 0.1492 | 1.106 (0.929 - 1.315) | 0.8652 |  | 0.8867 |
| TGM2 |  | 1.236 (0.971 - 1.574) | 0.2883 | 1.301 (1.025 - 1.652) | 0.1724 | 1.156 (0.931 - 1.435) | 0.8652 |  | 0.9103 |
| ACE2 |  | 1.498 (1.043 - 2.150) | 0.1955 | 1.299 (0.942 - 1.790) | 0.3046 | 1.218 (0.919 - 1.616) | 0.8652 |  | 0.8786 |
| CCL18 |  | 1.225 (0.890 - 1.687) | 0.4115 | 1.195 (0.914 - 1.564) | 0.3957 | 1.432 (1.075 - 1.907) | 0.5591 |  | 0.8564 |
| CD93 |  | 1.834 (1.037 - 3.245) | 0.213 | 1.342 (0.785 - 2.296) | 0.4935 | 1.592 (1.002 - 2.531) | 0.8107 |  | 0.8867 |
| IL1RL2 |  | 0.659 (0.391 - 1.111) | 0.3107 | 0.591 (0.354 - 0.986) | 0.2102 | 0.721 (0.486 - 1.069) | 0.8652 |  | 0.9337 |
| MMP-2 |  | 1.333 (0.814 - 2.183) | 0.4604 | 1.560 (0.951 - 2.557) | 0.2525 | 1.519 (1.011 - 2.282) | 0.8107 |  | 0.9475 |
| PGLYRP1 |  | 1.267 (0.873 - 1.840) | 0.4115 | 1.463 (1.052 - 2.035) | 0.1541 | 1.286 (0.942 - 1.755) | 0.8652 |  | 0.9171 |
| CCL14 |  | 1.592 (0.975 - 2.602) | 0.2584 | 1.645 (1.069 - 2.530) | 0.1541 | 1.241 (0.824 - 1.871) | 0.8652 |  | 0.8528 |
| Notch 3 |  | 1.274 (0.815 - 1.990) | 0.4841 | 1.730 (1.074 - 2.784) | 0.1541 | 1.442 (0.952 - 2.184) | 0.8606 |  | 0.8598 |
| OSMR |  | 2.901 (0.946 - 8.891) | 0.2584 | 2.848 (1.106 - 7.334) | 0.1724 | 1.523 (0.665 - 3.491) | 0.8652 |  | 0.8492 |
| CCL24 |  | 1.338 (1.024 - 1.748) | 0.2047 | 1.414 (1.105 - 1.808) | 0.1071 | 1.017 (0.819 - 1.264) | 0.9655 |  | 0.6912 |
| TIMP4 |  | 1.400 (0.916 - 2.140) | 0.3107 | 1.555 (1.020 - 2.370) | 0.2036 | 1.327 (0.915 - 1.923) | 0.8652 |  | 0.9394 |
| TRAIL-R2 |  | 1.833 (1.154 - 2.912) | 0.1475 | 1.856 (1.227 - 2.810) | 0.0788 | 1.055 (0.783 - 1.420) | 0.9412 |  | 0.596 |
| UMOD |  | 0.399 (0.173 - 0.922) | 0.2027 | 0.740 (0.342 - 1.603) | 0.6409 | 0.482 (0.236 - 0.986) | 0.8107 |  | 0.8492 |
| CD163 |  | 1.592 (0.979 - 2.589) | 0.2584 | 1.596 (0.991 - 2.572) | 0.2175 | 1.218 (0.831 - 1.786) | 0.8652 |  | 0.8528 |
| ICAM1 |  | **3.099 (1.622 - 5.924)** | **0.0342** | 1.456 (0.863 - 2.456) | 0.3661 | 1.074 (0.677 - 1.704) | 0.9554 |  | 0.596 |
| IL-27 |  | 2.050 (1.172 - 3.588) | 0.1475 | **2.641 (1.556 - 4.483)** | **0.0297** | 0.897 (0.616 - 1.306) | 0.886 |  | 0.4303 |
| OPN |  | 1.558 (1.126 - 2.155) | 0.1475 | 1.311 (0.930 - 1.846) | 0.323 | 1.113 (0.851 - 1.454) | 0.8656 |  | 0.7668 |
| RARRES2 |  | 2.584 (1.307 - 5.109) | 0.1475 | 1.048 (0.557 - 1.972) | 0.9359 | 1.799 (0.973 - 3.329) | 0.8459 |  | 0.6912 |
| SERPINA5 |  | 0.856 (0.535 - 1.370) | 0.6695 | 0.494 (0.315 - 0.775) | 0.0769 | 0.820 (0.554 - 1.212) | 0.8652 |  | 0.7173 |
| LTBP2 |  | 1.276 (0.721 - 2.259) | 0.5749 | 2.076 (1.210 - 3.563) | 0.1198 | 1.290 (0.867 - 1.919) | 0.8652 |  | 0.7671 |
| CRTAC1 |  | 0.863 (0.582 - 1.279) | 0.6193 | 0.562 (0.384 - 0.824) | 0.0782 | 0.854 (0.627 - 1.164) | 0.8652 |  | 0.7195 |
| GH |  | 1.014 (0.887 - 1.158) | 0.896 | 1.195 (1.050 - 1.360) | 0.1198 | 1.100 (0.981 - 1.234) | 0.8652 |  | 0.7668 |
| IGFBP-1 |  | 1.015 (0.844 - 1.221) | 0.9186 | 1.258 (1.058 - 1.495) | 0.1198 | 1.161 (0.984 - 1.371) | 0.8606 |  | 0.7668 |

Logistic model adjusted for sex, age, study drug and significant factors from Table 1

**Supplementary Table 5: Associations of protein biomarkers (significantly associated with overall CV events) with specific clinical events (MI/stroke, SCD, HF rehospitalization) using sIPTW.**

|  | **MI/Stroke** | | **SCD** | | **Rehospitalization** | | **Interaction** |
| --- | --- | --- | --- | --- | --- | --- | --- |
| **Biomarker** |  |  |  |  | **for HF** | | **FDR** |
|  | **OR (95% CI)** | **FDR** | **OR (95% CI)** | **FDR** | **OR (95% CI)** | **FDR** |  |
| NT-proBNP | 1.255 (1.081 - 1.457) | 0.10 | **1.426 (1.235 - 1.647)** | **0.0002** | **1.252 (1.113 - 1.408)** | **0.0491** | 0.7251 |
| BNP | 1.236 (1.086 - 1.407) | 0.0735 | **1.375 (1.217 - 1.553)** | **<.0001** | 1.197 (1.084 - 1.322) | 0.0505 | 0.6653 |
| TIMD4 | **2.898 (1.750 - 4.799)** | **0.0049** | 1.858 (1.273 - 2.713) | 0.0608 | 1.278 (0.918 - 1.779) | 0.8726 | 0.4328 |
| FGF-23 | 1.196 (0.989 - 1.446) | 0.2806 | 1.276 (1.091 - 1.491) | 0.0723 | 1.213 (1.050 - 1.402) | 0.4932 | 0.9241 |
| GDF-15 | 1.336 (1.003 - 1.780) | 0.2613 | 1.584 (1.191 - 2.105) | 0.0608 | 1.290 (0.993 - 1.676) | 0.8726 | 0.7922 |
| PSP-D | 2.043 (1.492 - 2.797) | 0.0023 | 1.434 (1.099 - 1.872) | 0.0883 | 1.110 (0.875 - 1.409) | 0.8726 | 0.3869 |
| SPON1 | 2.292 (1.361 - 3.860) | 0.0835 | 1.862 (1.145 - 3.028) | 0.0886 | 1.654 (1.060 - 2.580) | 0.7373 | 0.8231 |
| VEGFD | 2.230 (1.290 - 3.857) | 0.1258 | 1.980 (1.224 - 3.202) | 0.0798 | 1.414 (0.974 - 2.051) | 0.8726 | 0.7251 |
| TNC | 1.568 (1.109 - 2.218) | 0.164 | **1.955 (1.373 - 2.786)** | **0.0141** | 1.184 (0.900 - 1.558) | 0.8726 | 0.54 |
| AOC3 | 1.876 (1.105 - 3.186) | 0.1665 | 1.346 (0.848 - 2.135) | 0.3798 | 1.802 (1.182 - 2.745) | 0.4247 | 0.7949 |
| IGFBP-7 | 1.544 (1.030 - 2.315) | 0.2216 | 1.757 (1.200 - 2.572) | 0.0757 | 1.225 (0.878 - 1.709) | 0.8726 | 0.7308 |
| IL-1RT1 | 1.869 (1.124 - 3.107) | 0.1665 | 1.683 (1.002 - 2.828) | 0.183 | 1.636 (1.031 - 2.597) | 0.8433 | 0.957 |
| TFF3 | 1.284 (0.924 - 1.785) | 0.3568 | 1.780 (1.253 - 2.527) | 0.0608 | 1.130 (0.840 - 1.519) | 0.8726 | 0.6367 |
| TIMP1 | 2.278 (1.329 - 3.906) | 0.10 | 1.735 (1.141 - 2.638) | 0.0883 | 1.211 (0.834 - 1.758) | 0.8726 | 0.6367 |
| U-PAR | 1.610 (1.078 - 2.405) | 0.1665 | 1.694 (1.159 - 2.476) | 0.0817 | 1.166 (0.814 - 1.672) | 0.8726 | 0.7251 |
| ST2 | 1.609 (1.116 - 2.322) | 0.164 | 1.479 (1.122 - 1.950) | 0.0798 | 1.173 (0.904 - 1.521) | 0.8726 | 0.7251 |
| TR | 1.279 (0.934 - 1.753) | 0.3403 | 1.369 (1.016 - 1.845) | 0.1624 | 1.362 (1.050 - 1.766) | 0.6838 | 0.9673 |
| OPG | 1.566 (0.976 - 2.515) | 0.2806 | 1.749 (1.056 - 2.895) | 0.1392 | 1.497 (0.960 - 2.336) | 0.8726 | 0.9434 |
| TFPI | 2.017 (1.202 - 3.384) | 0.164 | 1.886 (1.083 - 3.284) | 0.1325 | 1.288 (0.809 - 2.051) | 0.8726 | 0.7447 |
| FCGR2A | 1.749 (1.161 - 2.634) | 0.164 | 1.286 (0.913 - 1.811) | 0.3209 | 1.295 (0.949 - 1.768) | 0.8726 | 0.757 |
| COL18A1 | 1.232 (0.799 - 1.899) | 0.5519 | 1.693 (1.124 - 2.550) | 0.0883 | 1.300 (0.896 - 1.886) | 0.8726 | 0.7831 |
| CXCL1 | 1.280 (1.040 - 1.575) | 0.1665 | 1.269 (1.038 - 1.552) | 0.1257 | 1.072 (0.894 - 1.286) | 0.8726 | 0.7308 |
| IGLC2 | 1.662 (1.106 - 2.497) | 0.1665 | 1.350 (0.942 - 1.935) | 0.2729 | 1.177 (0.839 - 1.652) | 0.8726 | 0.757 |
| IGFBP-2 | 1.213 (0.904 - 1.628) | 0.4346 | 1.514 (1.151 - 1.990) | 0.0723 | 1.086 (0.834 - 1.413) | 0.8832 | 0.6653 |
| IL6 | 1.189 (0.968 - 1.460) | 0.3079 | 1.247 (1.057 - 1.472) | 0.0883 | 1.111 (0.940 - 1.313) | 0.8726 | 0.8231 |
| TGM2 | 1.235 (0.986 - 1.547) | 0.2806 | 1.295 (1.031 - 1.627) | 0.1347 | 1.130 (0.926 - 1.379) | 0.8726 | 0.8253 |
| ACE2 | 1.564 (1.111 - 2.201) | 0.164 | 1.295 (0.958 - 1.751) | 0.2576 | 1.145 (0.890 - 1.473) | 0.8726 | 0.7308 |
| CCL18 | 1.220 (0.901 - 1.652) | 0.4346 | 1.215 (0.941 - 1.569) | 0.3037 | 1.428 (1.079 - 1.888) | 0.5792 | 0.8253 |
| CD93 | 1.631 (0.967 - 2.752) | 0.2806 | 1.347 (0.818 - 2.219) | 0.423 | 1.455 (0.945 - 2.240) | 0.8726 | 0.9328 |
| IL1RL2 | 0.672 (0.406 - 1.113) | 0.3403 | 0.610 (0.370 - 1.003) | 0.183 | 0.759 (0.525 - 1.098) | 0.8726 | 0.8951 |
| MMP-2 | 1.333 (0.839 - 2.118) | 0.4539 | 1.582 (0.998 - 2.508) | 0.183 | 1.438 (0.980 - 2.108) | 0.8726 | 0.9328 |
| PGLYRP1 | 1.176 (0.831 - 1.664) | 0.5631 | 1.496 (1.095 - 2.044) | 0.0883 | 1.242 (0.916 - 1.685) | 0.8726 | 0.7926 |
| Notch 3 | 1.224 (0.812 - 1.846) | 0.5465 | 1.751 (1.120 - 2.735) | 0.096 | 1.414 (0.944 - 2.118) | 0.8726 | 0.7789 |
| OSMR | 3.312 (1.115 - 9.840) | 0.2183 | 3.003 (1.231 - 7.328) | 0.1057 | 1.376 (0.623 - 3.039) | 0.8726 | 0.7251 |
| CCL24 | 1.323 (1.029 - 1.701) | 0.2104 | 1.441 (1.134 - 1.832) | 0.0723 | 1.028 (0.837 - 1.262) | 0.9457 | 0.54 |
| IGFBP-1 | 1.006 (0.842 - 1.202) | 0.9518 | 1.249 (1.065 - 1.465) | 0.0817 | 1.158 (0.987 - 1.358) | 0.8726 | 0.6585 |
| CD163 | 1.529 (0.977 - 2.393) | 0.2806 | 1.673 (1.058 - 2.646) | 0.1358 | 1.225 (0.845 - 1.777) | 0.8726 | 0.7922 |
| MMP-9 | 1.087 (0.897 - 1.316) | 0.5912 | 1.255 (1.059 - 1.485) | 0.0883 | 1.115 (0.938 - 1.324) | 0.8726 | 0.7678 |
| **ICAM1** | **3.350 (1.790 - 6.269)** | **0.0144** | 1.482 (0.899 - 2.443) | 0.2827 | 1.030 (0.661 - 1.606) | 0.9551 | 0.3869 |
| OPN | 1.485 (1.096 - 2.012) | 0.164 | 1.300 (0.937 - 1.804) | 0.2827 | 1.078 (0.832 - 1.398) | 0.8985 | 0.7216 |
| SERPINA5 | 0.859 (0.550 - 1.341) | 0.6674 | 0.517 (0.337 - 0.794) | 0.0723 | 0.816 (0.563 - 1.184) | 0.8726 | 0.6585 |
| LTBP2 | 1.278 (0.737 - 2.215) | 0.5788 | 2.084 (1.262 - 3.440) | 0.0757 | 1.255 (0.853 - 1.848) | 0.8726 | 0.7043 |
| CRTAC1 | 0.860 (0.592 - 1.250) | 0.632 | 0.600 (0.421 - 0.854) | 0.0793 | 0.857 (0.637 - 1.153) | 0.8726 | 0.7043 |
